# Evaluation of pharmacological and pharmacokinetic herb-drug interaction between irinotecan hydrochloride injection and Kangai injection in colorectal tumor-bearing mice and healthy rats

**DOI:** 10.3389/fphar.2023.1282062

**Published:** 2023-11-29

**Authors:** Yanfei Chen, Zhaoliang Hu, Jing Jiang, Chenxi Liu, Shuxiao Gao, Min Song, Taijun Hang

**Affiliations:** ^1^ School of Hainan Provincial Drug Safety Evaluation Research Center, Hainan Medical University, Haikou, China; ^2^ Key Laboratory of Drug Quality Control and Pharmacovigilance (China Pharmaceutical University), Ministry of Education, Nanjing, China

**Keywords:** herb-drug interaction, irinotecan, Kangai injection, colorectal cancer, pharmacology, pharmacokinetics

## Abstract

**Introduction:** Kangai (KA) injection, a Chinese herbal injection, is often used in combination with irinotecan (CPT-11) to enhance the effectiveness of anti-colorectal cancer treatment and alleviate side effects. However, the combined administration of this herb-drug pair remains controversial due to limited pre-clinical evidence and safety concerns. This study aimed to determine the pre-clinical herb-drug interactions between CPT-11 and KA injection to provide a reference for their clinical co-administration.

**Methods:** In the pharmacological study, BALB/c mice with CT26 colorectal tumors were divided into four groups and treated with vehicle alone (0.9% saline), CPT-11 injection (100 mg/kg), KA injection (10 mL/kg), or a combination of CPT-11 and KA injection, respectively. The tumor volume of mice was monitored daily to evaluate the therapeutic effect. Daily body weight, survival rate, hematopoietic toxicity, immune organ indices, and gut toxicity were analyzed to study the adverse effects. Healthy Sprague-Dawley rats in the pharmacokinetic study were administered KA injection only (4 mL/kg), or a combination of CPT-11 injection (20 mg/kg) and KA injection, respectively. Six key components of KA injection (oxymatrine, matrine, ginsenoside Rb1, Rg1, Re, and astragaloside IV) in rat plasma samples collected within 24 h after administration were determined by LC-MS/MS.

**Results:** The pharmacological study indicated that KA injection has the potential to enhance the anti-colorectal cancer efficacy of CPT-11 injection and alleviate the severe weight loss induced by CPT-11 injection in tumor-bearing mice. The pharmacokinetic study revealed that co-administration resulted in inhibition of oxymatrine metabolism in rats, evidenced by the significantly reduced C_max_ and AUC_0-t_ of its metabolite, matrine (*p* < 0.05), from 2.23 ± 0.24 to 1.38 ± 0.12 μg/mL and 8.29 ± 1.34 to 5.30 ± 0.79 μg h/mL, respectively. However, due to the similar efficacy of oxymatrine and matrine, this may not compromise the anti-cancer effect of this herb-drug pair.

**Discussion:** This study clarified the pre-clinical pharmacology and pharmacokinetic benefits and risks of the CPT-11-KA combination and provided a reference for their clinical co-administration.

## 1 Introduction

Combining Chinese herbal drugs with conventional single-target medicines often enhances clinical efficacy and reduces toxicity when treating complex diseases such as cancer (Li and Weng, 2017; Sun et al., 2019; Liu et al., 2021). For example, irinotecan (CPT-11) is a commonly used chemotherapy for the treatment of colorectal cancer (de Man et al., 2018; National Comprehensive Cancer Network, 2023). However, adverse effects such as nausea and vomiting, leukopenia, weight loss, and diarrhea are often induced by CPT-11 during colorectal cancer treatment (National Comprehensive Cancer Network, 2023). Therefore, Kangai (KA) injection, an herbal anti-cancer injection approved by the China National Medical Products Administration, is typically used as a supplement and adjuvant therapy in combination with CPT-11 for the treatment of colorectal cancer to enhance clinical efficacy and reduce toxicity (Jiang and Zhu, 2011; Li, 2014; Cai et al., 2015; Huang et al., 2019).

Kangai (KA) injection is composed of oxymatrine (OMT), *ginseng* (*Panax ginseng* C.A.Mey.), and *astragalus* (*Astragalus membranaceus* (Fisch.) Bunge) extracts. OMT is the main component of *sophora* (*Sophora flavescens* Aiton), and our previous research indicated that it accounts for the highest proportion of the KA injection, reaching 83% (9,249 μg/mL) (Chen Y. et al., 2021). The remaining 17% of the KA injection is made up of *ginseng* and *astragalus* extracts, containing 2.67 μg/mL of ginsenoside Rb1 (Rb1), 83.8 μg/mL of ginsenoside Rg1 (Rg1), 60.1 μg/mL of ginsenoside Re (Re), and 81.5 μg/mL of astragaloside IV (AS-IV) (Chen Y. et al., 2021). Li conducted a clinical study with 97 patients on the co-administration of the KA injection and FOLFIRI, which is composed of CPT-11 as the main component, combined with leucovorin and fluorouracil (Li, 2014; Huang et al., 2019; National Comprehensive Cancer Network, 2023). The result indicated that after co-administration with the KA injection, the overall response rate (ORR) to FOLFIRI increased from 41.7% to 59.2% (*p* < 0.05), the Karnofsky performance status (KPS) score increased from 63.7 ± 4.5 to 69.6 ± 5.1, and the rate of nausea and vomiting decreased from 35.4% to 10.2% (Li, 2014; Huang et al., 2019). Another clinical study of 60 patients conducted by Jiang and Zhu indicated that after co-administration with the KA injection, the KPS score increased from 63.3% to 90.0%, and the rate of leukopenia and nausea and vomiting decreased from 30.0% to 6.67% and 46.7% to 10.0%, respectively (Jiang and Zhu, 2011; Huang et al., 2019). These clinical studies have demonstrated that the addition of the KA injection can enhance the efficacy of anti-colorectal cancer treatment and alleviate the side effects of the chemotherapy regimen with CPT-11 as the main component. However, the rationality and safety of the combined administration of CPT-11 and KA injection remain controversial due to the lack of comprehensive pre-clinical evidence. Therefore, pre-clinical pharmacology studies need to be conducted to illustrate how KA injection affects the anti-colorectal cancer efficacy and side effects of CPT-11.

CPT-11 is a water-soluble analog of camptothecin (de Man et al., 2018). CPT-11 has two significant metabolic pathways: one is activated by carboxylesterases (CEs) and produces metabolite SN-38 (7-ethyl-10-hydroxycamptothecin) and the other is inactivated by cytochrome P450 3A (CYP3A) and results in the formation of metabolite APC 7-ethyl-10-(4-N-aminopentanoicacid)-1-piperidino)carbonyloxycamptothecin (de Man et al., 2018; Paulik et al., 2018). Because OMT is metabolized to MT primarily via CYP3A4, CPT-11 may compete with OMT for this enzyme, thereby altering its pharmacokinetic parameters (Liu et al., 2015). Therefore, pre-clinical pharmacokinetic interaction studies between CPT-11 and the KA injection are crucial. The results can help predict clinical pharmacokinetic changes and corresponding risks.

Based on the above urgent needs, this study aimed to explore the pharmacological impact of KA injection on the anti-tumor efficacy and side effects of CPT-11 using a CT26 colorectal cancer-bearing BALB/c mouse model and to elucidate the pharmacokinetic interaction of the herb-drug pair in healthy rats. Side effects induced by CPT-11 discussed in this study include body weight loss, hematopoietic toxicity, immune organ atrophy, and gut toxicity. Because our previous studies have shown that the KA injection does not cause pharmacokinetic changes in CPT-11 and its metabolites in rat plasma, this study only focused on the effects of CPT-11 on the pharmacokinetics of key components in the KA injection (Chen Y. et al., 2021). The findings are expected to clarify the pre-clinical pharmacology and pharmacokinetic benefits and risks of the CPT-11-KA combination and provide a reference for their clinical co-administration.

## 2 Materials and methods

### 2.1 Reagents and medications

Kangai injection (10 and 20 mL, lot numbers: 01170635, 01180409, and 02190309) was procured from Changbaishan Pharmaceutical Co., Ltd. (Jilin, China). Irinotecan hydrochloride injection (100 per 5 mL, lot number: 17022731) was donated by Hengrui Medicine Co. Ltd. (Jiangsu, China). Analytical grade reagents (acetic acid, ammonium acetate, and formic acid) and HPLC grade methanol were obtained from Nanjing Chemical Reagent Co. Ltd. (Nanjing, China) and Tedia Company Inc. (Fairfield, OH, United States), respectively. The chemical reference substances for OMT, Re, and donepezil were obtained from Aladdin (Shanghai, China), the National Institutes for Food and Drug Control (Beijing, China), and Luoxin Pharmaceutical Group Stock Co. Ltd. (Linyi, China), respectively. Matrine (MT), Rb1, Rg1, AS-IV, and digoxin were purchased from Desite Biological Technology Co. Ltd. (Chengdu, China). RPMI 1640 medium, penicillin-streptomycin (Pen-Strep), and fetal bovine serum (FBS) were obtained from Gibco (Grand Island, NY, United States), Beyotime Biotechnology (Shanghai, China), and Biological Industries (Kibbutz Beit-Haemek, Israel), respectively. Hematology diluent, 4% paraformaldehyde, paraffin, and Hematoxylin and Eosin (H&E) were purchased from Mindray (Shenzhen, China), Biosharp (Hefei, China), Citotest Scientific Co., Ltd. (Haimen, China), and Sigma-Aldrich (St. Louis, MO, United States), respectively.

### 2.2 Cell lines

The CT26 mouse colon adenocarcinoma cell line was obtained from the Chinese National Collection of Authenticated Cell Cultures (Shanghai, China) and cultured in RPMI 1640 medium supplemented with 1% Pen-Strep and 10% FBS at 37°C with 5% CO_2_.

### 2.3 Anti-colorectal cancer efficacy study

#### 2.3.1 Mouse tumor model and treatments

Male BALB/c mice (6 weeks old; body weight: 20–26 g) were obtained from SIPPR-Bk Lab Animal Co., Ltd. (Shanghai, China). The mice were housed in the Laboratory Animal Center of China Pharmaceutical University at 20°C–24°C with 30%–70% relative humidity and acclimated for 7 days before the experiment. All experiments in this study were approved by the Animal Ethics Committee of China Pharmaceutical University (permission No. 201911002).

The CT-26 cells (0.2 mL of 1 × 10^5^) suspended in 0.2 mL of PBS were injected subcutaneously into the right flank of each mouse. When tumor volume reached approximately 100 mm^3^ (estimated by length × width^2^ × 0.5), the mice were divided into four groups (*n* = 7). Group 1 was treated with the vehicle alone (0.9% saline), Group 2 with CPT-11 injection, Group 3 with KA injection, and Group 4 with a combination of CPT-11 and KA injection. According to the guidance of the U.S. Food and Drug Administration, the animal dosage was converted from the human dosage using the following formula: Animal dose (mg/kg) = Human dose (mg/kg) × (Human K_m_/Animal K_m_) (U. S. Food and Drug Administration, 2005). For KA injection, the drug standard and label information indicated that the dose for humans is 40–60 mL per day; therefore, the KA injection dose for mice should be 8.26–12.3 mL/kg. Thus, 10 mL/kg KA injection was intraperitoneally (i.p.) administered to mice every day, containing 92.5 mg/kg of OMT, 0.03 mg/kg of Rb1, 0.84 mg/kg of Rg1, 0.60 mg/kg of Re, and 0.82 mg/kg of AS-IV. Similarly, based on the clinical dose of 125 mg/m^2^ per day, the dose of CPT-11 injection for mice should be 40 mg/kg (The United States National Library of Medicine, 2022). However, similar to other studies, our previous study showed that no significant suppression effect of CPT-11 on tumor growth in tumor-bearing mice was observed at this dose, possibly due to the differences between species (Liu et al., 2019; Chattopadhyay et al., 2020). To observe the herb-drug interaction better, mice were weekly i. p. administered with the maximum tolerated dose (MTD) of CPT-11 injection, which is 100 mg/kg (Cao et al., 2004). A schematic diagram of the administration method is shown in Figure 2A. Following 17 days of treatment, all mice were euthanized by cervical dislocation.

#### 2.3.2 Hematopoietic toxicity study

At the end of the treatment, 40 μL of blood was collected from the orbital sinus of tumor-bearing mice and diluted with 140 μL of hematology diluent. The samples were run on an XN-1000 Hematology Analyzer (Sysmex Corporation, Kobe, Japan) to analyze hematological parameters.

#### 2.3.3 Thymus and spleen indices analysis

After tumor-bearing mice were euthanized, the thymus and spleen were collected, washed with cold saline, and gently blotted dry with filter paper. The thymus or spleen weight of each mouse was then divided by the total body weight on the last day to calculate the thymus or spleen indices according to the formula: thymus or spleen indices (%) = weight of thymus or spleen (g)/weight of mouse (g).

#### 2.3.4 Gut toxicity study

CPT-11-induced diarrhea was monitored by placing a clean white paper on the bottom of the cage on the 2nd, 9th, and 16th days after the first administration. The severity of diarrhea was assessed according to the following criteria: 0: dry normal stool; 1: slightly soft and wet stool; 2: moderately stained perianal coat with unformed stool; 3: severely stained perianal coat with watery stool (Kurita et al., 2000). Duodenum and colon samples were collected and fixed in 4% paraformaldehyde after euthanizing all mice. Tissue samples were then dehydrated in alcohol, cleared using xylene, embedded in paraffin wax, and subsequently sectioned. Following H&E staining, photomicrographs of the tissue sections were captured for histopathological examination.

### 2.4 Pharmacokinetic study

#### 2.4.1 Instrumentation and conditions

**FIGURE 1:**
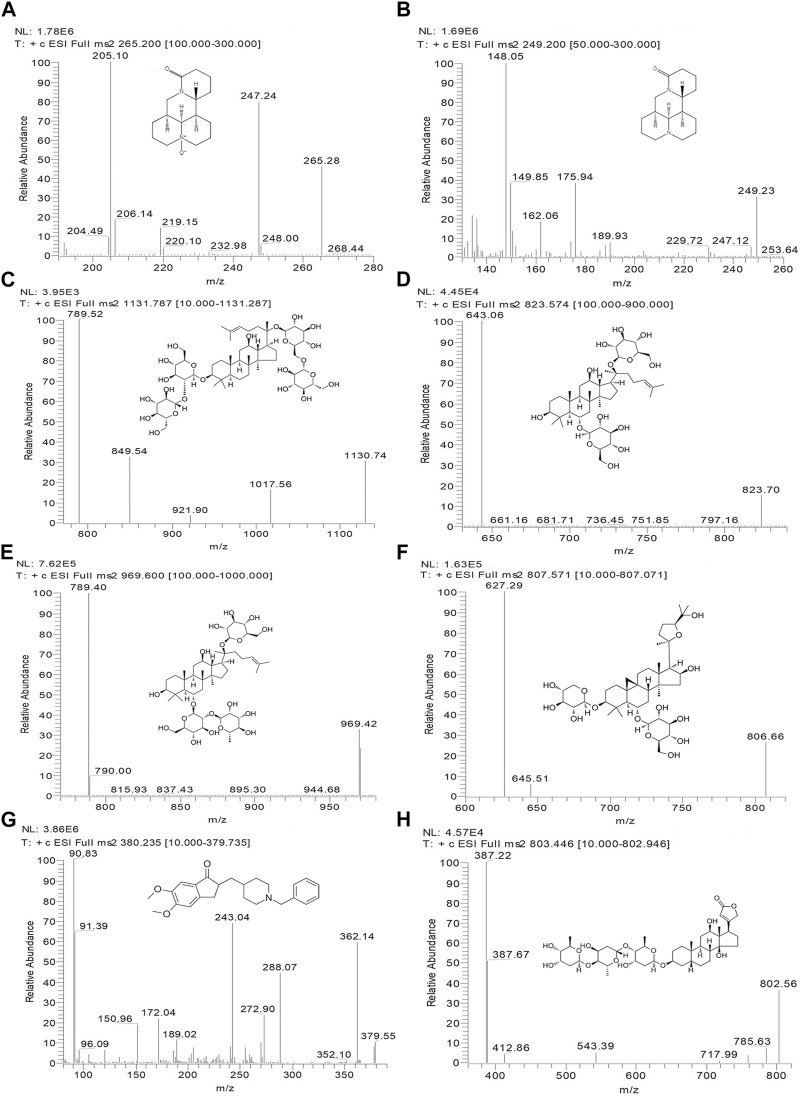
Chemical structures and MS/MS product ion spectra for key components of KA injection. OMT **(A)**, MT **(B)**, Rb1 **(C)**, Rg1 **(D)**, Re **(E)**, AS-IV **(F)**, donepezil (IS) **(G)**, and digoxin (IS) **(H)**.

In this study, a Thermo Dionex Ultimate 3000 HPLC system coupled to a TSQ Quantum Ultra AM triple quadrupole mass spectrometer with an electrospray ionization (ESI) source (Thermo Fisher Scientific, MA, United Statesa) was used to analyze all biological samples. OMT and MT were separated in isocratic mode with a mobile phase of a methanol-water solution (70:30) containing 0.2% acetic acid and 0.2% ammonium acetate. Separation was achieved by using a Phenomenex Luna 5u CN 100R (250*4.6 mm, 5 μm) column at 35°C with a flow rate of 0.8 mL/min and an injection volume of 20 μL. Rb1, Rg1, Re, and AS-IV were separated on an Inertsil C8-3 (150* 4.6 mm, 5 μm) column at 35°C. The mobile phase comprised a methanol-water solution (5:95) containing 0.1% formic acid (A) and a 0.1% formic acid methanol solution (B) with a gradient elution program (A: B): 0.0 min (50: 50) → 6.0 min (20: 80) → 9.0 min (20: 80) → 9.1 min (50: 50) → 10.1 min (50: 50). The injection volume and flow rate were 20 μL and 1.0 mL/min, respectively. Key parameters of the mass spectrometer, including the capillary temperature, ion spray voltage, nitrogen sheath gas, and auxiliary gas, were optimized at 350°C, 4000 V, 275 kPa, and 35 kPa, respectively, and operated in positive mode. The multiple-reaction monitoring (MRM) acquisition mode was employed to monitor the ion transitions of the key components within the KA injection. The product ion spectra and specific MRM parameters are shown in Figure 1 and Table 1, respectively.

**TABLE 1:** The MRM parameters for the key components of KA injection.

Compound	Precursor ion (m/z)	Product ion (m/z)	Collision energy (eV)
OMT	265.3	205.1	28
MT	249.2	148.0	30
Rb1	1,130.7	789.5	40
Rg1	823.7	643.1	33
Re	969.4	789.4	40
AS-IV	806.7	627.3	38
Donepezil (IS)	379.6	90.8	30
Digoxin (IS)	802.6	387.2	43

#### 2.4.2 Animals and treatments in pharmacokinetics

Healthy Sprague-Dawley (SD) rats (8–12 weeks old; body weight: 180–230 g) were purchased from SIPPR-Bk Lab Animal Co., Ltd. (Shanghai, China) and raised in the Laboratory Animal Center of China Pharmaceutical University. Twelve SD rats were divided into two groups with three males and three females in each group and acclimated for 7 days prior to pharmacokinetic analyses. Group 1 received vehicle (0.9% saline) and Group 2 was treated with CPT-11 injection. After 15 min, the KA injection was administered to SD rats in both groups. According to the clinical dose and the conversion formula based on the guidance by the U.S. Food and Drug Administration, 20 mg/kg CPT-11 injection and 4 mL/kg KA injection (containing 37.0 mg/kg of OMT, 0.01 mg/kg of Rb1, 0.34 mg/kg of Rg1, 0.24 mg/kg of Re, and 0.33 mg/kg of AS-IV) were intravenously administered to rats (U. S. Food and Drug Administration, 2005; The United States National Library of Medicine, 2022). All rats were intravenously injected with an equal volume of 5 mL/kg.

Blood samples (approximately 0.2 mL) were obtained from the retro-orbital venous plexus and added to heparin-containing tubes at 0, 0.083, 0.25, 0.5, 1, 2, 4, 8, 12, and 24 h following the administration of KA injection. Saline was provided every 2 h to promote the recovery of the blood volume of rats. Blood samples were centrifuged at 1,000 *g* for 10 min to separate the plasma and then stored at −20°C until required.

#### 2.4.3 Bio-assays

A mixture containing 50 µL of plasma sample, 50 µL of internal standard (IS), 100 µL of methanol, and 100 μL of methanol with 0.1% formic acid was vortexed for 3 min and then centrifuged at 16,000 × *g* force for 10 min. The upper layer was evaporated *in vacuo* to dry at 37°C, followed by reconstitution in 150 μL of 80% methanol. After further centrifugation at 16,000 × *g* force for 10 min, 20 µL of the supernatant was collected for the LC-MS/MS analysis.

#### 2.4.4 Preparation of calibration standards and quality controls (QC)

Stock solutions of OMT, MT, Rb1, Rg1, Re, and AS-IV were prepared individually in methanol at a concentration of 200 μg/mL. The stock solutions of alkaloids (OMT and MT) and saponins (Rb1, Rg1, Re, and AS-IV) were mixed separately and serially diluted with methanol to obtain working solutions. Similarly, the working solutions of the IS (donepezil and digoxin) were prepared at 200 ng/mL and 20 μg/mL, respectively. Calibration standards were established by adding working solutions of alkaloids and saponins into blank rat plasma to obtain concentrations ranging from 10 to 4,000 ng/mL. Quality control (QC) samples were prepared the same as the calibration standards at 20, 1,000, and 3,000 ng/mL for alkaloids and 20, 1,000, and 3,200 ng/mL for saponins.

#### 2.4.5 Method validation

The bioanalytical method was validated by assessing the following elements: selectivity, linearity, accuracy, precision, matrix effects, recovery, and stability.

### 2.5 Data analysis

All data are expressed as means ± standard deviation (SD). In the pharmacological study, BALB/c mice with CT26 colorectal tumors were divided into four groups (*n* = 7). Tumor volume and body weight were compared between groups using a two-way ANOVA test in SPSS software (version 24.0, SPSS Inc., Chicago, IL, United States). One-way ANOVA test with Bonferroni *post hoc* test was used to compare data on tumor weight, hematopoietic toxicity, and thymus and spleen indices between multiple groups. Diarrhea scores were compared using the Mann-Whitney *U* test. Survival analysis was performed via the log-rank test (Mantel-Cox) in Prism software (GraphPad Software, San Diego, CA, United States). In the pharmacokinetic study, rats were assigned to two groups (*n* = 6). Plasma pharmacokinetic parameters were computed using WinNonlin 6.2 (Pharsight, St. Louis, MO, United States), and comparisons between the two groups were assessed by unpaired Student’s t-test using SPSS software. A *p*-value less than 0.5 was considered statistically significant.

## 3 Results

### 3.1 Determination of the anti-colorectal cancer efficacy of CPT-11 and KA injection

**FIGURE 2:**
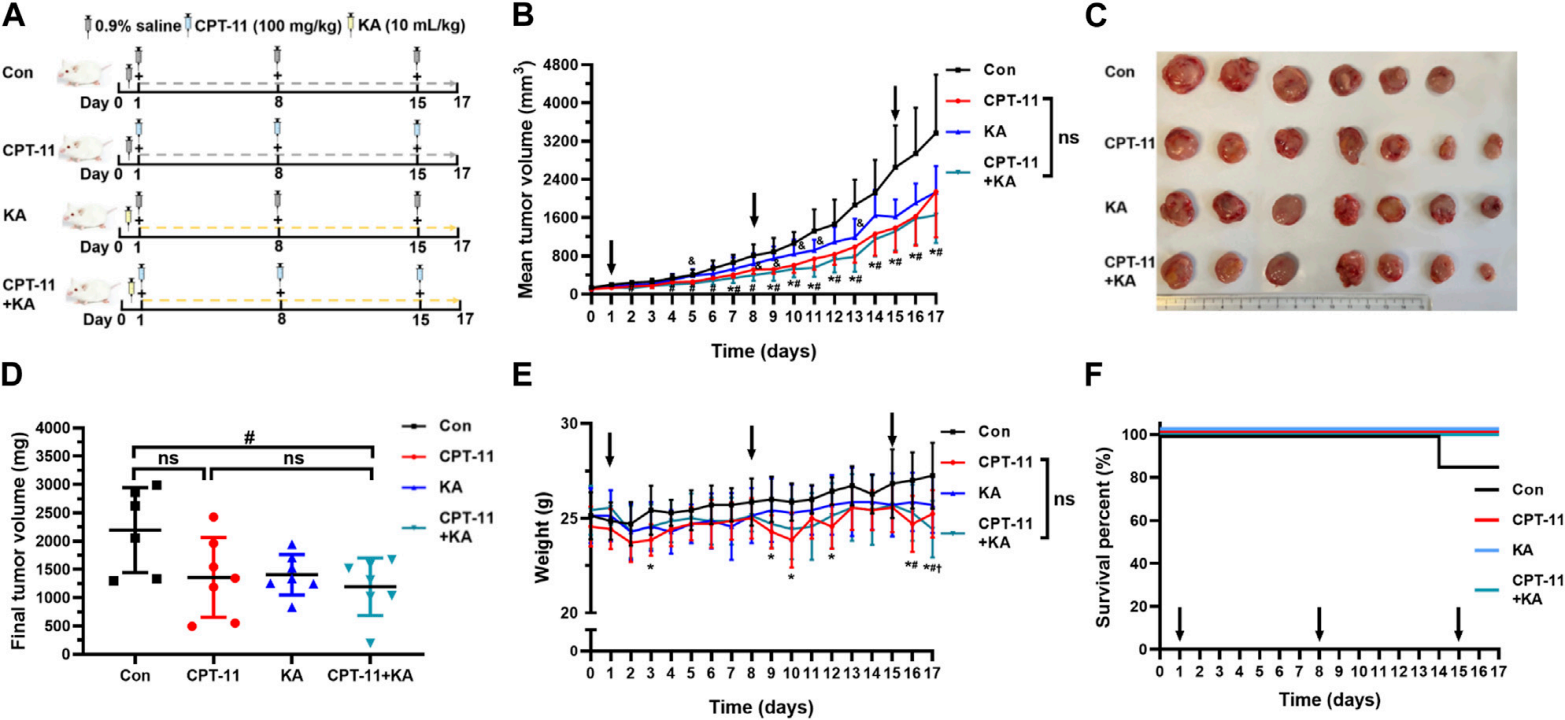
Co-administration of CPT-11 with KA injection better inhibited tumor growth and alleviated weight loss in CT-26 tumor-bearing mice (*n* = 7). Mice were treated with vehicle (saline) alone, 100 mg/kg CPT-11 injection weekly, 10 mL/kg KA injection daily, or a combination of CPT-11 (100 mg/kg) and KA (10 mL/kg). Schematic diagram of the study design **(A),** tumor volume **(B)**, macroscopic images of tumors **(C)**, tumor weights **(D)**, body weight **(E)**, and survival percent curves **(F)**. Data are presented as mean ± SD. Statistical Analysis: two-way ANOVA test **(B)** and **(E)**, one-way ANOVA test with Bonferroni *post hoc* test **(D)**, and log-rank test (Mantel-Cox) **(F)**. ns: not significant; *, *p* < 0.05, CPT-11 vs. Con; †, *p* < 0.05, KA vs. Con; #, *p* < 0.05, CPT-11+KA vs. Con; &, *p* < 0.05, KA vs. CPT-11+KA.

In comparison to the control (Con) group, CPT-11 injection showed anti-tumor activity on the 7th and 9–17th day (*p* < 0.05) (Figure 2B). The co-administration group experienced a significant reduction in tumor volume compared to the Con group on the second, and 4–17th day (*p* < 0.05), exhibiting earlier and longer tumor reduction than the CPT-11 injection group. Furthermore, while the final tumor weight in the CPT-11 injection group did not show a significant difference compared to the Con group, it was notably reduced in the co-administration group (*p* < 0.05) (Figure 2D). Therefore, although no clear differences were observed between the co-administration group and the CPT-11 injection group, based on the above results, the KA injection showed the potential to enhance the anti-cancer efficacy of CPT-11. Compared to the KA injection group, the tumor volume in the co-administration group was significantly reduced on the 5th, 8–11th, and 13th day (*p* < 0.05), which supports the potential synergistic anti-tumor effect of this herb-drug pair from another perspective. Macroscopic images of tumors presented in Figure 2C underpin these findings.

The body weight of tumor-bearing mice was monitored to assess the safety of the medicines. In the CPT-11 injection group, compared to the Con group, mice showed severe weight loss within 1, 2 days following CPT-11 injection (*p* < 0.05), notably on the 3rd, 9th, 10th, 12th, 16th, and 17th days (Figure 2E). Conversely, such significant weight reduction was not observed in the other groups. The KA injection group and co-administration group exhibited a more reduced body weight than the Con group only in the last 1–2 days of the treatment period. At this time, the weight of the Con group was higher than that of all treatment groups. This may be because the Con group did not receive treatment, resulting in excessively large tumors and a correspondingly higher weight in the late stage of cancer. Therefore, although no clear differences were observed between the single-agent and the co-administration group, KA injection may have the potential to alleviate severe weight loss induced by CPT-11 injection. Throughout the treatment and observation period, only one mouse in the Con group died on the 14th day. As shown in Figure 2F, there was no significant difference in survival benefit among the groups (*p* > 0.05).

### 3.2 Reduction of CPT-11 injection-induced hematopoietic toxicity by KA injection

**FIGURE 3:**
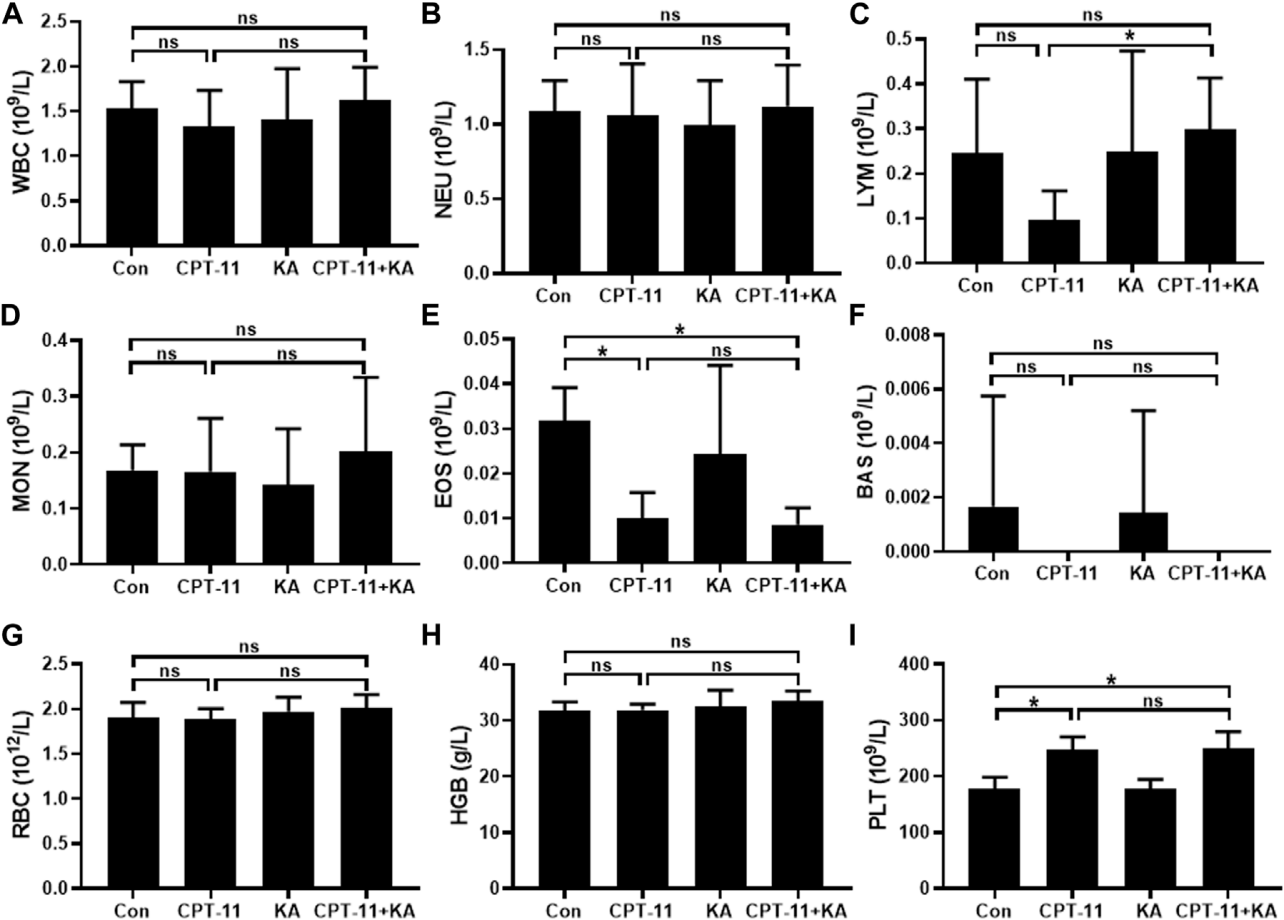
Representative hematological parameters (n = 7). White blood cells **(A)**, neutrophils **(B)**, lymphocytes **(C)**, monocytes **(D)**, eosinophils **(E)**, basophils **(F)**, red blood cells **(G)**, hemoglobin **(H)**, and platelets **(I)**. Data are presented as mean ± SD. Statistical Analysis: one-way ANOVA test with Bonferroni *post hoc* test. ns: not significant; *p < 0.05.

The results of hematopoietic toxicity following treatment are shown in Figure 3. Mice treated with CPT-11 and KA injection demonstrated significantly higher lymphocyte counts compared with the CPT-11 injection group (*p* < 0.05) (Figure 3C). Neither the CPT-11 injection group nor the co-administration group showed statistical difference from the Con group. Furthermore, both CPT-11 injection and co-administration groups had lower blood eosinophil counts than that of the Con group (*p* < 0.01), but no difference was observed between the single-agent and the co-administration group (Figure 3E). In addition, compared to the Con group, the platelet count was significantly increased after CPT-11 injection treatment (*p* < 0.001) (Figure 3I), and co-administration with KA injection had no obvious effects on it. Regardless of treatment, no other hematological parameters were significantly changed.

### 3.3 Effect of KA injection on CPT-11 injection-induced immune organ atrophy

**FIGURE 4:**
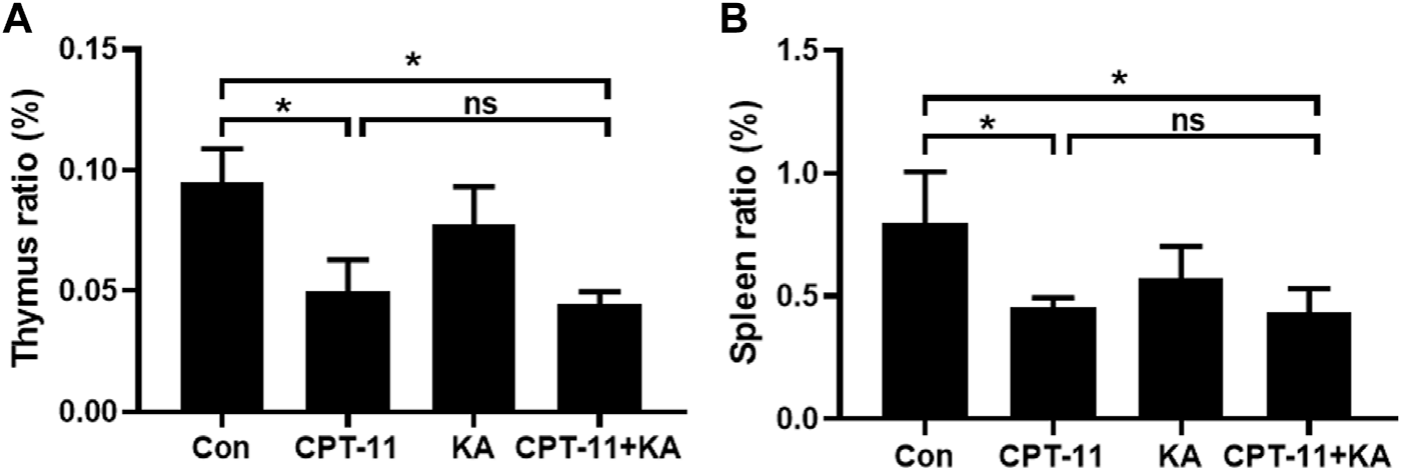
KA injection was unable to relieve CPT-11-induced immunosuppression of CT-26 tumor-bearing mice (n = 7). The thymus **(A)** and spleen **(B)** indices. Data are presented as mean ± SD. Statistical Analysis: one-way ANOVA test with Bonferroni *post hoc* test. ns: not significant; *p < 0.05.

As shown in Figure 4, CPT-11 injection reduced both the thymus and spleen indices of mice. However, KA injection did not relieve the immune organ atrophy, and no statistical difference was observed between the CPT-11 injection group and the co-administration group.

### 3.4 Effect of KA injection on CPT-11 injection-induced gut toxicity

**FIGURE 5:**
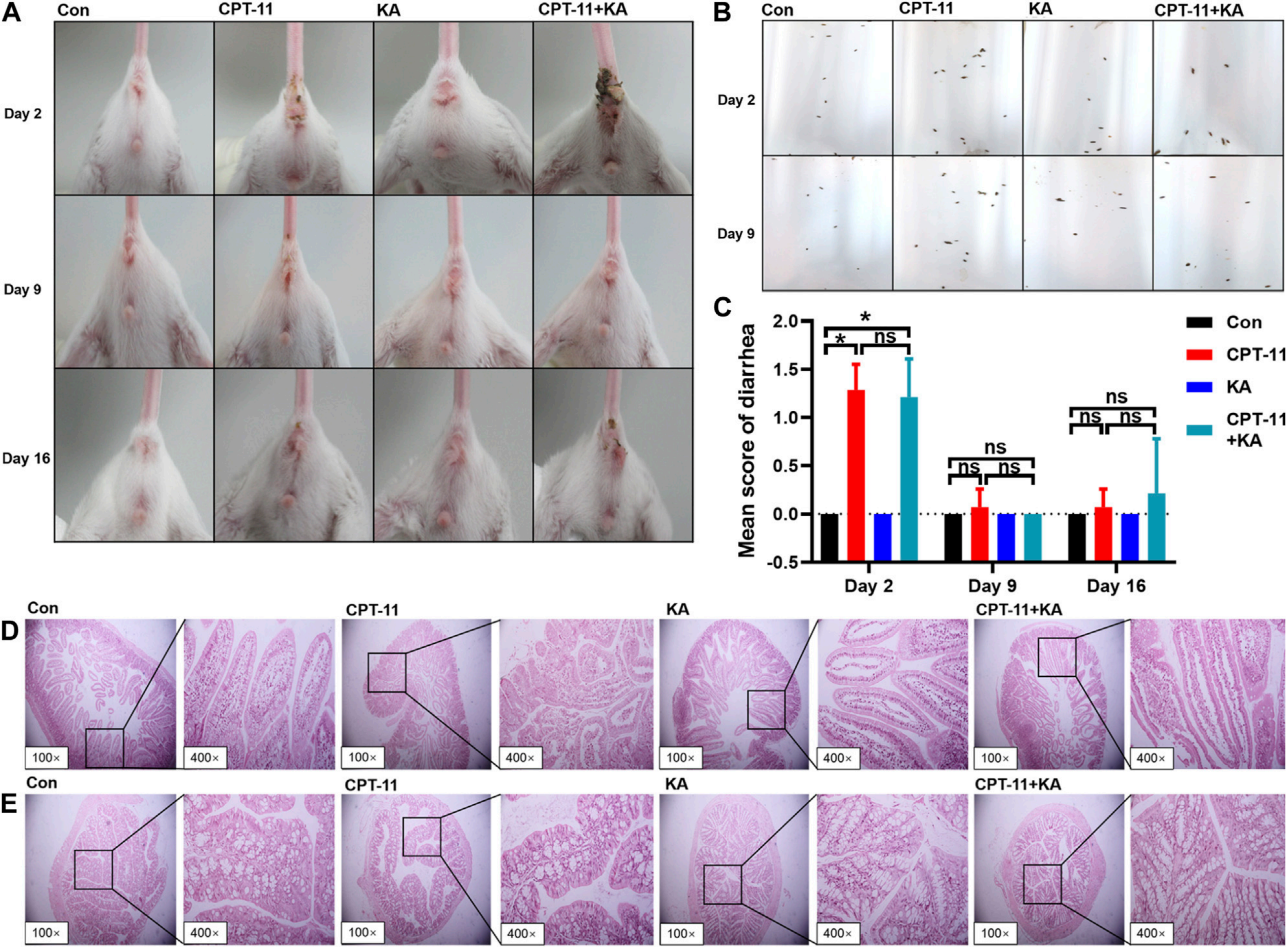
KA injection was unable to attenuate CPT-11-induced gut toxicity of CT-26 tumor-bearing mice after treatment (*n* = 7). The photographs of the perianal coat **(A)** and stool **(B)**, the score of diarrhea **(C)**, and the histological sections of the duodenum **(D)** and colon **(E)**. Data are presented as mean ± SD. Statistical Analysis: Mann-Whitney *U* test **(C)**. ns: not significant; *p < 0.05.

Images of the perianal coat, stool, and delayed-onset diarrhea score at 24 h after treatment are shown in Figures 5A–C, respectively. In comparison to the Con group, the CPT-11 injection group experienced diarrhea (*p* < 0.05) and showed a moderately stained perianal coat with soft and wet stool. Co-administration with KA injection did not protect mice from diarrhea. As shown in Figure 5D, histological changes in the duodenum after the CPT-11 injection administration were observed, with disordered intestinal cell arrangement, marked degeneration and necrosis of mucosal epithelial cells, and thinning of the intestinal wall, indicative of intestinal injury. However, the group treated with both CPT-11 and the KA injection did not demonstrate a reduction in tissue injury. Regardless of treatment, the colon showed healthy tissue morphology with intact mucosa and no inflammatory cell infiltration or necrosis (Figure 5E). In conclusion, KA injection was unable to attenuate CPT-11-induced gut toxicity.

### 3.5 Pharmacokinetic method validation

**FIGURE 6:**
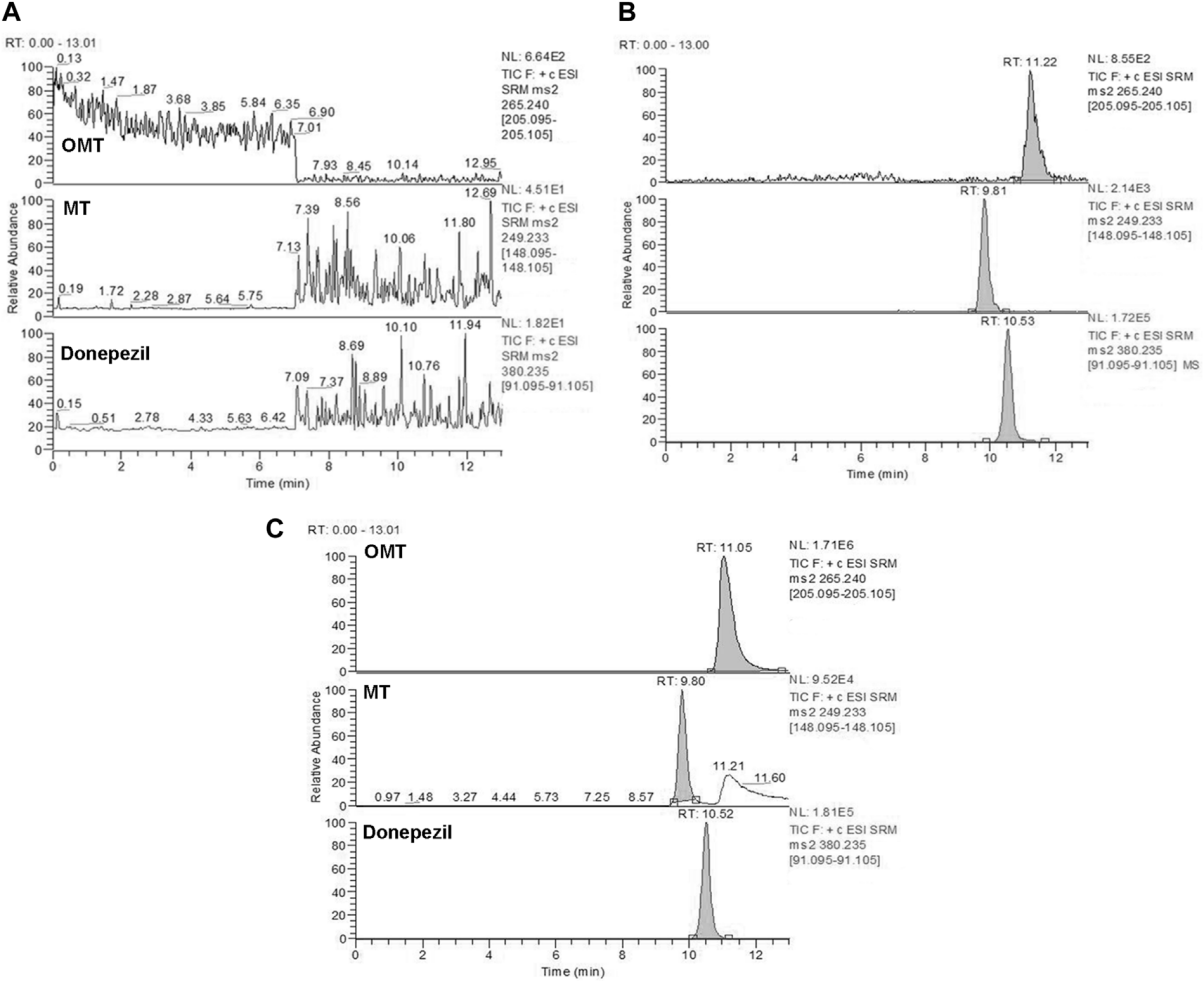
Representative MRM chromatograms for OMT and MT. Blank plasma samples **(A)**, blank plasma samples spiked with OMT (10 ng/mL), MT (10 ng/mL), and donepezil (IS) (200 ng/mL) **(B)**, and rat plasma samples collected 5 min after treatment of CPT-11 and KA injection containing OMT (35646 ng/mL) and MT (607 ng/mL) **(C)**. Retention time: OMT (11.2 min), MT (9.81 min) and donepezil (IS) (10.5 min).

**FIGURE 7:**
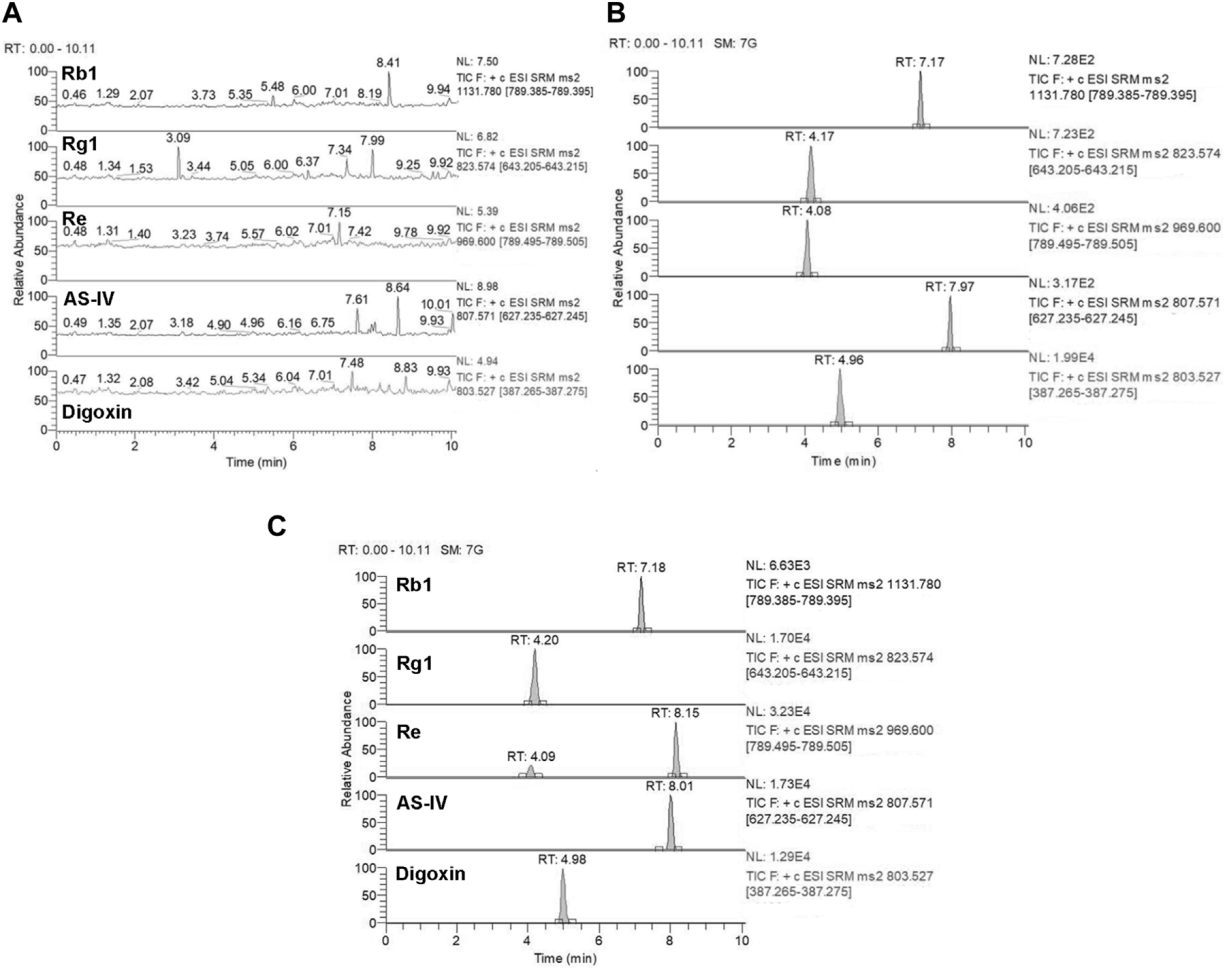
Representative MRM chromatograms for Rb1, Rg1, Re, and AS-IV. Blank plasma samples **(A)**, blank plasma samples spiked with Rb1 (10 ng/mL), Rg1 (10 ng/mL), Re (10 ng/mL), AS-IV (10 ng/mL), and digoxin (IS) (20 μg/mL) **(B)**, and rat plasma samples collected 5 min after treatment of CPT-11 and KA injection containing Rb1 (163 ng/mL), Rg1 (422 ng/mL), Re (373 ng/mL), and AS-IV (854 ng/mL) **(C)**. Retention time: Rb1 (7.17 min), Rg1 (4.17 min), Re (4.08 min), AS-IV (7.97 min), and digoxin (IS) (4.96 min).

Selectivity: Based on the results presented in Figures 6, 7, the bioanalytical method was selective for analyzing the analytes in plasma. No significant interference was noticed at the retention times corresponding to the key components of KA injection.

Linearity and lower limits of quantification (LLOQ): Representative calibration curves and the LLOQ for the key components of KA injection are summarized in Supplementary Table S1. The correlation coefficients (R) all exceeded 0.99. The LLOQ was sufficient for the bioanalysis conducted in this study.

Accuracy and precision: Supplementary Table S2 lists the accuracy and precision data for the bioanalytical method. All data conformed to the criteria outlined by the ICH for bioassays.

Matrix effect and recovery: As shown in Supplementary Table S3, the matrix effect of plasma was not significant and the method exhibited sufficiently high recovery rates.

Stability: Stability data for the key components of KA injection are listed in Supplementary Table S4. No notable degradation was observed under various conditions, indicating the stability of all analytes during the experiment.

### 3.6 Effect of CPT-11 on the pharmacokinetics of KA injection

**FIGURE 8:**
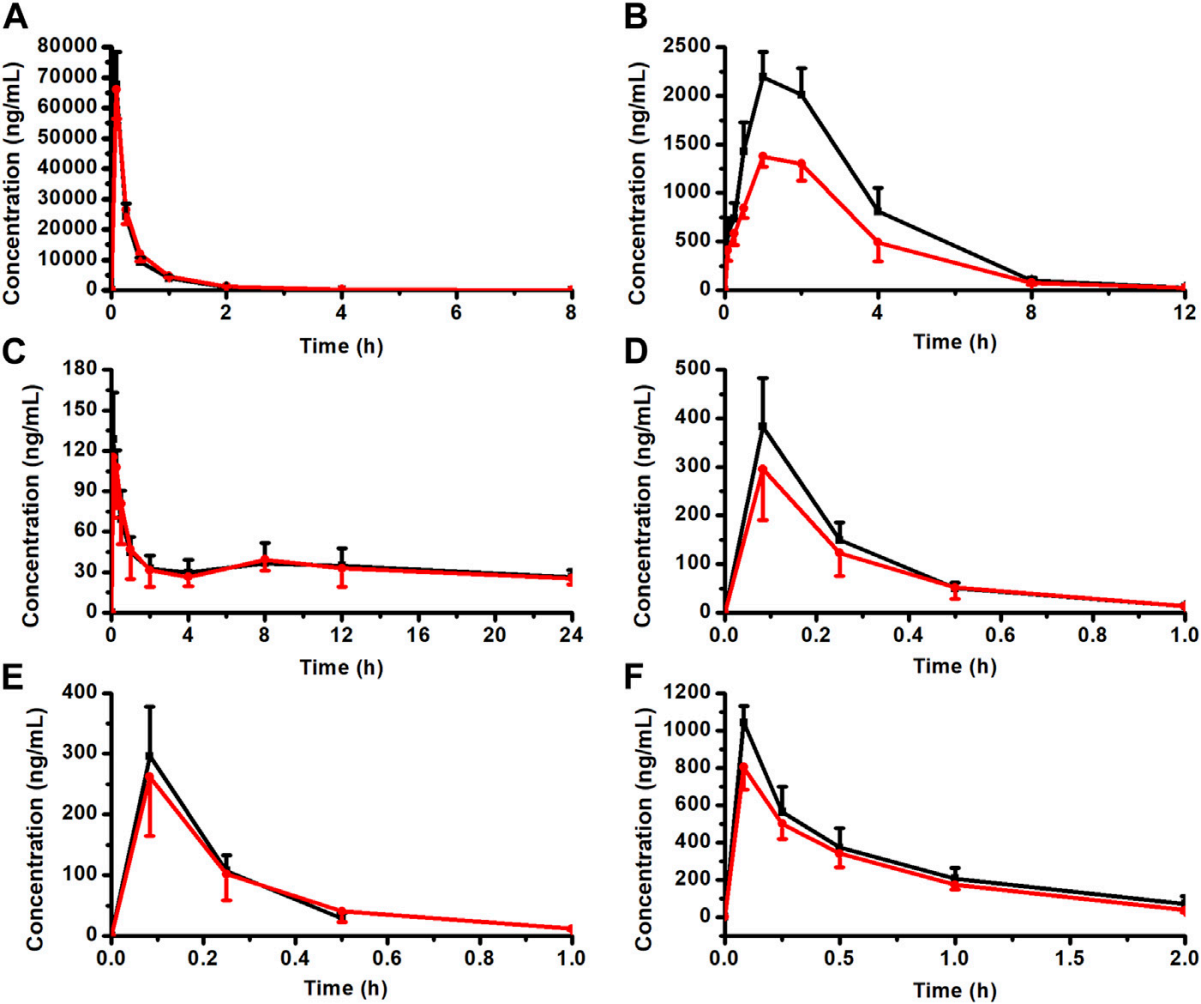
Plasma concentration-time profiles for the key components of KA injection after the single administration and co-administration with CPT-11 injection (*n* = 6). OMT **(A)**, MT **(B)**, Rb1 **(C)**, Rg1 **(D)**, Re **(E)**, and AS-IV **(F)**. 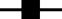
KA injection group, 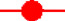
CPT-11 + KA injection co-administration group.

The rat plasma pharmacokinetic profile of OMT, MT, Rb1, Rg1, Re, and AS-IV in KA injection after co-administration with CPT-11 was studied using the validated LC-MS/MS method. The plasma concentration-time profiles are shown in Figure 8, and the corresponding pharmacokinetic parameters are listed in Tables 2, 3. Compared with the KA injection group, the MRT_0-t_ for OMT of the co-administration group was significantly extended, and the C_max_, AUC_0-t_, and AUC_0-∞_ of its metabolite, MT, were reduced (*p* < 0.05), which indicates the inhibition of OMT metabolism. Furthermore, the C_0_ of AS-IV was significantly decreased after co-administration. Pharmacokinetic parameters of other key components in KA injection showed no significant changes following co-administration with CPT-11 injection.

**TABLE 2:** The plasma pharmacokinetic parameters for the key alkaloids of KA injection after the single administration and co-administration with CPT-11 injection.

PK parameters	OMT		MT	
	KA	CPT-11+KA	KA	CPT-11+KA
T_max_ (h)	NA	NA	1.33 ± 0.52	1.33 ± 0.52
C_0_ (ng/mL)	67590 ± 10909	65983 ± 9,491	NA	NA
C_max_ (ng/mL)	NA	NA	2,229 ± 243	1,383 ± 117^*^
AUC_0-t_ (ng·h/mL)	21566 ± 2,558	24282 ± 2,697	8,290 ± 1,338	5,298 ± 793^*^
AUC_0-∞_ (ng·h/mL)	21639 ± 2,541	24308 ± 2,703	8,345 ± 1,355	5,367 ± 801^*^
t_1/2_ (h)	0.83 ± 0.22	0.95 ± 0.13	1.55 ± 0.09	1.9 ± 0.42
MRT_0-t_ (h)	0.50 ± 0.05	0.60 ± 0.05^*^	2.57 ± 0.16	2.64 ± 0.22
V_d_ (mL/kg)	2050 ± 457	2097 ± 307	NA	NA
CL (mL/h/kg)	1733 ± 235	1,537 ± 157	NA	NA

*p* < 0.05, compared with the KA, injection group.

NA: not applicable.

**TABLE 3:** The plasma pharmacokinetic parameters for the key saponins of KA injection after the single administration and co-administration with CPT-11 injection.

PK parameters	Rb1		Rg1		Re		AS-IV	
	KA	CPT-11+KA	KA	CPT-11+KA	KA	CPT-11+KA	KA	CPT-11+KA
C_0_ (ng/mL)	133 ± 32	135 ± 28	383 ± 100	296 ± 105	296 ± 81	262 ± 97	1,043 ± 86	803 ± 120^*^
AUC_0-t_ (ng·h/mL)	547 ± 346	611 ± 232	91.7 ± 18.1	83.6 ± 31.0	63.0 ± 14.7	66.4 ± 24.8	579 ± 107	481 ± 35
AUC_0-∞_ (ng·h/mL)	2,811 ± 3,975	1,654 ± 334	103 ± 9.92	97.4 ± 24.4	NC	NC	647 ± 158	509 ± 23
t_1/2_ (h)	52.0 ± 88.4	23.2 ± 10.6	0.22 ± 0.05	0.24 ± 0.04	NC	NC	0.61 ± 0.13	0.49 ± 0.14
MRT_0-t_ (h)	7.03 ± 4.32	7.45 ± 3.05	0.20 ± 0.05	0.25 ± 0.05	0.17 ± 0.01	0.22 ± 0.06	0.57 ± 0.06	0.55 ± 0.04
V_d_ (mL/kg)	214 ± 75	220 ± 91	1,041 ± 333	1,307 ± 537	NC	NC	449 ± 48	456 ± 147
CL (mL/h/kg)	15.7 ± 20.3	6.69 ± 1.36	3,261 ± 313	3,633 ± 983	NC	NC	527 ± 117	642 ± 30

*p* < 0.05, compared with the KA, injection group.

NC: not calculated.

## 4 Discussion

Kangai injection is typically combined with CPT-11 for clinical colorectal cancer treatment (Jiang and Zhu, 2011; Li, 2014; Cai et al., 2015; Huang et al., 2019). However, the validity of this combination has been questioned due to the lack of pre-clinical studies assessing its potential benefits and risks. Therefore, this study continued our previous research on the herb-drug interaction between CPT-11 and KA injection. We aimed to scientifically evaluate the pre-clinical pharmacological impact of the KA injection on the anti-tumor efficacy and side effects of CPT-11 and the effects of CPT-11 on the pharmacokinetics of key components of the KA injection. Referring to previous studies, the herb-drug interactions were evaluated by comparing co-administration and single-agent administration rather than by comparing with positive drugs (Guan et al., 2017; Razmovski-Naumovski et al., 2022).

The pharmacological result of BALB/c mice with CT26 colorectal tumors indicated that although no clear difference in tumor volume was observed between the CPT-11 injection group and the co-administration group, KA injection advanced and extended the tumor suppression time of CPT-11. Furthermore, while the final tumor weight in the CPT-11 injection group did not show a significant difference compared to the Con group, it was notably reduced in the co-administration group (*p* < 0.05). Based on the above results, the KA injection showed the potential to enhance the anti-cancer efficacy of CPT-11. Compared with the KA injection group, the tumor volume in the co-administration group was significantly reduced (*p* < 0.05), which may also support the potential synergistic anti-tumor effect of this herb-drug pair from another perspective. No clear difference in body weight was observed between the CPT-11 injection group and the co-administration group. However, compared with the Con group, mice showed severe weight loss within 1, 2 days following CPT-11 injection (*p* < 0.05), whereas this was not observed in the co-administration group. Therefore, KA injection may have the potential to alleviate severe weight loss induced by CPT-11 injection. The efficacy of KA injection in enhancing the anti-colorectal cancer efficacy of CPT-11 and alleviating CPT-11-induced weight loss may be related to the bioactive components in the KA injection. The KA injection is composed of OMT (the main component of *sophora*), *ginseng*, and *astragalus* extract. In traditional Chinese medicine (TCM), *sophora* is frequently used in the treatment of ulcerative colitis, a major cause of colorectal cancer (Chen et al., 2020; Shah and Itzkowitz, 2022). Recently, researchers demonstrated that the key components of KA injection, such as OMT and its metabolite MT have the potential to inhibit colorectal cancer progression and enhance the effects of chemotherapies by inducing cell apoptosis and inhibiting proliferation (Duan et al., 2017; Liang et al., 2020; Chen M. et al., 2021; Pan et al., 2021). A meta-analysis of 1,145 patients indicated that compound kushen injection, which mainly contains oxymatrine and matrine, improved the anti-cancer efficacy of chemotherapeutic drugs and KPS and reduced the incidence of nausea, vomiting, and leucopenia in patients with colorectal cancer (Yu et al., 2017). *Astragalus* and *ginseng* have been well-known tonics in TCM for a long time and recently showed potential in cancer treatment (Zuo et al., 2022). This effect may be attributed to the active components in *astragalus* such as astragalin and AS-IV that inhibit the proliferation and migration of cancer cells (Li et al., 2020; Yang et al., 2021). Some bioactive components in *ginseng*, such as protopanaxatriol saponins, can also prevent the development of colorectal cancer in animals (Majeed et al., 2018; Wu et al., 2022; Wang et al., 2023). A meta-analysis of 1,409 participants showed that *astragalus*-based medicines combined with chemotherapy may improve the tumor response rate and KPS and reduce incidences of nausea, vomiting, and neutropenia during the treatment of colorectal cancer (Lin et al., 2019). Another meta-analysis involving 334,544 participants from 1990 to 2014 indicated that patients treated with *ginseng* had a significantly reduced risk of developing cancer (16%) (Jin et al., 2016).

Although co-administration with the KA injection in mice conferred potential protection against adverse effects caused by CPT-11, its efficacy remains limited. For example, the KA injection did not protect CPT-11-treated mice from diarrhea and intestinal injury. Furthermore, CPT-11 reduced thymus and spleen indices in tumor-bearing mice, which are important indicators of the level of immune regulation (Zhang et al., 2022). Co-administration with the KA injection did not mitigate CPT-11-induced atrophy of immune organs. Therefore, although lymphocyte counts in the CPT-11 injection group were significantly increased after co-administration with the KA injection (*p* < 0.05), monitoring the immune function of a patient using the herb-drug pair in clinical treatment is crucial.

Hematopoietic toxicity results indicated that both the CPT-11 injection and co-administration groups had a lower blood eosinophil count than that of the Con group (*p* < 0.01). Eosinophilia is associated with many gastrointestinal disorders, such as inflammatory bowel disease, which can increase the risk of colorectal cancer (Sakkal et al., 2016). Therefore, both CPT-11 injection and co-administration therapy may inhibit the development of colorectal cancer. CPT-11 injection led to increased platelet count, which may exacerbate cancer metastasis, but the co-administration with KA injection showed no obvious effects (Sylman et al., 2017). Therefore, physicians and health professionals should monitor platelet counts and CPT-11-induced gut toxicity in patients using this herb-drug pair to treat colorectal cancer.

In pre-clinical pharmacokinetic studies, rats appear to be the most commonly used species (Bahloul et al., 2018). Furthermore, rats have larger circulating blood volume than mice; thus, sufficient plasma can be obtained with fewer animals, which is consistent with the 3R (replacement, reduction, and refinement) principle (Diehl et al., 2001; Bahloul et al., 2018). Therefore, this study used rats for pharmacokinetic studies. The results showed that compared with the KA injection group, the MRT_0-t_ of OMT was extended (*p* < 0.05) and the C_max_ and AUC of its metabolite MT were reduced (*p* < 0.05) in the co-administration group, indicating that the metabolism of OMT was inhibited. CPT-11 may competitively inhibit the metabolism of OMT because it is another important substrate of CYP3A4, the main metabolic enzyme of OMT (Liu et al., 2015; de Man et al., 2018). However, previous studies indicated that both OMT and MT have the potential to suppress colorectal cancer progression and enhance the effect of chemotherapy, and the results of the current pharmacological study demonstrated that the KA injection showed the potential to enhance the anti-cancer efficacy of CPT-11 (Duan et al., 2017; Liang et al., 2020; Chen M. et al., 2021; Pan et al., 2021). Therefore, the metabolic inhibition of OMT may not affect the anti-colorectal cancer efficacy of this herb-drug pair. Furthermore, the C_0_ of AS-IV was significantly reduced, but other pharmacokinetic parameters remained constant. Although no pharmacodynamic changes associated with this variation were identified in this study, physicians still need to monitor this aspect in long-term clinical treatment. Mice and humans possess a gall bladder but rats do not; therefore, the pharmacokinetic behavior may be different from these species (Bahloul et al., 2018). Thus, further studies on the pharmacokinetic behavior in other species should be performed to reproduce our results. The results can be collectively used to guide clinical pharmacokinetic studies.

## 5 Conclusion

This study further develops our previous herb-drug interaction research of the CPT-11-KA injection combination, showing that the KA injection has the potential to enhance the anti-cancer efficacy of CPT-11 and alleviate the severe weight loss induced by CPT-11 injection in BALB/c mice with CT26 colorectal tumors. However, co-administration with the KA injection did not protect mice from some adverse side effects caused by CPT-11, such as gut toxicity and immune organ atrophy. Although metabolic inhibition of OMT in the KA injection was observed in healthy rats after co-administration, the anti-colorectal cancer efficacy of this herb-drug pair may remain unaffected. In conclusion, this study clarifies the pre-clinical benefits of the CPT-11-KA combination and provides support and reference for their clinical co-administration. However, this study also indicates the risks of the herb-drug pair. Considering the continued presence of side effects induced by CPT-11 and uncommon adverse drug reactions that may be caused by the complex chemical composition of the KA injection, physicians should monitor adverse reactions related to this herb-drug pair during long-term clinical treatment for safety and enhanced efficacy.

## Data Availability

The original contributions presented in the study are included in the article/Supplementary Material, further inquiries can be directed to the corresponding author.

## Abbreviations

APC, 7-ethyl-10-(4-N-aminopentanoicacid)-1-piperidino)carbonyloxycamptothecin; AS-IV, astragaloside IV; Con, control; CPT-11, irinotecan; CYP3A, cytochrome P450 3A; FBS, fetal bovine serum; FOLFIRI, Folinic acid + Fluorouracil + Irinotecan; IS, internal standard; KA, Kangai; KPS, Karnofsky performance status; LLOQ, lower limits of quantification; MRM, multiple-reaction-monitoring; MT, matrine; MTD, maximum tolerated dose; OMT, oxymatrine; ORR, overall response rate; Pen-Strep, penicillin-streptomycin; QC, quality control; Rb1, ginsenoside Rb1; Re, ginsenoside Re; Rg1, ginsenoside Rg1; RPMI, Roswell Park Memorial Institute; SD, Sprague-Dawley; SD, standard deviation; SN-38, 7-ethyl-10-hydroxycamptothecin; TCM, traditional Chinese medicine.

## References

[B1] Bahloul B. Safta F. Lassoued M. Dhotel H. Seguin J. Mignet N. (2018). Use of mouse model in pharmacokinetic studies of poorly water soluble drugs: application to fenofibrate. J. Drug Deliv. Sci. Technol. 43, 149–153. 10.1016/j.jddst.2017.10.006

[B2] Cai D. Jiang X. Lin G. (2015). Efficacy of kangai injection combined with chemotherapy in 90 patients with advanced colon cancer. J. North Pharm. 12, 184.

[B3] Cao S. Durrani F. Rustum Y. (2004). Selective modulation of the therapeutic efficacy of anticancer drugs by selenium containing compounds against human tumor xenografts. Clin. Cancer Res. 10, 2561–2569. 10.1158/1078-0432.ccr-03-0268 15073137

[B4] Chattopadhyay S. Liu Y. H. Fang Z. S. Lin C. L. Lin J. C. Yao B. Y. (2020). Synthetic immunogenic cell death mediated by intracellular delivery of STING agonist nanoshells enhances anticancer chemo-immunotherapy. Nano Lett. 20, 2246–2256. 10.1021/acs.nanolett.9b04094 32160474

[B5] Chen M. Ding Y. Tong Z. (2020). Efficacy and safety of Sophora flavescens (kushen) based traditional Chinese medicine in the treatment of ulcerative colitis: clinical evidence and potential mechanisms. Front. Pharmacol. 11, 603476. 10.3389/fphar.2020.603476 33362558 PMC7758483

[B6] Chen M. Gu Y. Zhang A. Sze D. Mo S. May B. (2021a). Biological effects and mechanisms of matrine and other constituents of Sophora flavescens in colorectal cancer. Pharmacol. Res. 171, 105778. 10.1016/j.phrs.2021.105778 34298110

[B7] Chen Y. Hu Z. Qi W. Gao S. Jiang J. Wang S. (2021b). Pharmacovigilance of herb-drug interactions: a pharmacokinetic study on the combination administration of herbal Kang’ai injection and chemotherapy irinotecan hydrochloride injection by LC–MS/MS. J. Pharm. Biomed. Anal. 194, 113784. 10.1016/j.jpba.2020.113784 33280996

[B8] de Man F. M. Goey A. K. L. van Schaik R. H. N. Mathijssen R. H. J. Bins S. (2018). Individualization of irinotecan treatment: a review of pharmacokinetics, pharmacodynamics, and pharmacogenetics. Clin. Pharmacokinet. 57, 1229–1254. 10.1007/s40262-018-0644-7 29520731 PMC6132501

[B9] Diehl K. H. Hull R. Morton D. Pfister R. Rabemampianina Y. Smith D. (2001). A good practice guide to the administration of substances and removal of blood, including routes and volumes. J. Appl. Toxicol. 21, 15–23. 10.1002/jat.727 11180276

[B10] Duan L. Deng L. Wang D. Ma S. Li C. Zhao D. (2017). Treatment mechanism of matrine in combination with irinotecan for colon cancer. Oncol. Lett. 14, 2300–2304. 10.3892/ol.2017.6407 28781667 PMC5530135

[B11] Guan H. Li P. Wang X. Yue J. He Y. Luo X. (2017). Shengjiang xiexin decoction alters pharmacokinetics of irinotecan by regulating metabolic enzymes and transporters: a multi-target therapy for alleviating the gastrointestinal toxicity. Front. Pharmacol. 8, 769. 10.3389/fphar.2017.00769 29163158 PMC5663900

[B12] Huang S. Peng W. Mao D. Zhang S. Xu P. Yi P. (2019). Kangai injection, a traditional Chinese medicine, improves efficacy and reduces toxicity of chemotherapy in advanced colorectal cancer patients: a systematic review and meta-analysis. Evid.-based Complement. Altern. Med. 2019, 8423037. 10.1155/2019/8423037 PMC6662435 31379968

[B13] Jiang Q. Zhu H. (2011). Clinical study of kangai injection combined with FORFIRI chemotherapy in the treatment of 30 cases of advanced colon cancer. J. Kunming. Med. Univ. 32, 52–53+56. 10.3969/j.issn.1003-4706.2011.09.014

[B14] Jin X. Che D. Zhang Z. Yan H. Jia Z. Jia X. (2016). Ginseng consumption and risk of cancer: a meta-analysis. J. Ginseng Res. 40, 269–277. 10.1016/j.jgr.2015.08.007 27616903 PMC5005362

[B15] Kurita A. Kado S. Kaneda N. Onoue M. Hashimoto S. Yokokura T. (2000). Modified irinotecan hydrochloride (CPT-11) administration schedule improves induction of delayed-onset diarrhea in rats. Cancer Chemother. Pharmacol. 46, 211–220. 10.1007/s002800000151 11021738

[B16] Li F. Weng J. (2017). Demystifying traditional herbal medicine with modern approach. Nat. Plants. 3, 17109. 10.1038/nplants.2017.109 28758992

[B17] Li K. (2014). Observation on kang'ai injection combined with chemotherapy for advanced colon cancer. J. Liaoning Univ. Tradit. Chin. Med. 16, 19–21. 10.13194/j.issn.1673-842x.2014.11.006

[B18] Li S. Sun Y. Huang J. Wang B. Gong Y. Fang Y. (2020). Anti-tumor effects and mechanisms of Astragalus membranaceus (AM) and its specific immunopotentiation: status and prospect. J. Ethnopharmacol. 258, 112797. 10.1016/j.jep.2020.112797 32243990

[B19] Liang L. Wu J. Luo J. Wang L. Chen Z. Han C. (2020). Oxymatrine reverses 5-fluorouracil resistance by inhibition of colon cancer cell epithelial-mesenchymal transition and NF-κB signaling *in vitro* . Oncol. Lett. 19, 519–526. 10.3892/ol.2019.11090 31897166 PMC6924048

[B20] Lin S. An X. Guo Y. Gu J. Xie T. Wu Q. (2019). Meta-analysis of astragalus-containing traditional Chinese medicine combined with chemotherapy for colorectal cancer: efficacy and safety to tumor response. Front. Oncol. 9, 749. 10.3389/fonc.2019.00749 31456940 PMC6700271

[B21] Liu W. Shi J. Zhu L. Dong L. Luo F. Zhao M. (2015). Reductive metabolism of oxymatrine is catalyzed by microsomal CYP3A4. Drug Des. devel. Ther. 9, 5771–5783. 10.2147/dddt.S92276 PMC4636097 26586934

[B22] Liu X. Jiang J. Chan R. Ji Y. Lu J. Liao Y. (2019). Improved efficacy and reduced toxicity using a custom-designed irinotecan-delivering silicasome for orthotopic colon cancer. ACS Nano 13, 38–53. 10.1021/acsnano.8b06164 30525443 PMC6554030

[B23] Liu Y. Chang M. Hu Z. Xu X. Wu W. Ning M. (2021). Danggui Buxue Decoction enhances the anticancer activity of gemcitabine and alleviates gemcitabine-induced myelosuppression. J. Ethnopharmacol. 273, 113965. 10.1016/j.jep.2021.113965 33639205

[B24] Majeed F. Malik F. Z. Ahmed Z. Afreen A. Afzal M. N. Khalid N. (2018). Ginseng phytochemicals as therapeutics in oncology: recent perspectives. Biomed. Pharmacother. 100, 52–63. 10.1016/j.biopha.2018.01.155 29421582

[B25] National Comprehensive Cancer Network (2023). NCCN clinical practice guidelines in oncology colon cancer. Available at: https://www.nccn.org/professionals/physician_gls/pdf/colon.pdf (Accessed November 08, 2023). 10.6004/jnccn.2009.0056 19755046

[B26] Pan D. Zhang W. Zhang N. Xu Y. Chen Y. Peng J. (2021). Oxymatrine synergistically enhances doxorubicin anticancer effects in colorectal cancer. Front. Pharmacol. 12, 673432. 10.3389/fphar.2021.673432 34305593 PMC8297828

[B27] Paulik A. Nekvindova J. Filip P. S. (2018). Irinotecan toxicity during treatment of metastatic colorectal cancer: focus on pharmacogenomics and personalized medicine. Tumori J. 106, 87–94. 10.1177/0300891618811283 30514181

[B28] Razmovski-Naumovski V. Kimble B. Laurenti D. Nammi S. Norimoto H. Chan K. (2022). Polysaccharide peptide extract from coriolus versicolor increased T(max) of tamoxifen and maintained biochemical serum parameters, with No change in the metabolism of tamoxifen in the rat. Front. Pharmacol. 13, 857864. 10.3389/fphar.2022.857864 35450034 PMC9016780

[B29] Sakkal S. Miller S. Apostolopoulos V. Nurgali K. (2016). Eosinophils in cancer: favourable or unfavourable? Curr. Med. Chem. 23, 650–666. 10.2174/0929867323666160119094313 26785997

[B30] Shah S. C. Itzkowitz S. H. (2022). Colorectal cancer in inflammatory bowel disease: mechanisms and management. Gastroenterology 162, 715–730.e3. 10.1053/j.gastro.2021.10.035 34757143 PMC9003896

[B31] Sun X. Xu X. Chen Y. Guan R. Cheng T. Wang Y. (2019). Danggui buxue decoction sensitizes the response of non-small-cell lung cancer to gemcitabine via regulating deoxycytidine kinase and P-glycoprotein. Molecules 24, 2011. 10.3390/molecules24102011 31130654 PMC6572355

[B32] Sylman J. L. Mitrugno A. Tormoen G. W. Wagner T. H. Mallick P. Mccarty O. J. T. (2017). Platelet count as a predictor of metastasis and venous thromboembolism in patients with cancer. Converg. Sci. Phys. Oncol. 3, 023001. 10.1088/2057-1739/aa6c05 29081989 PMC5658139

[B33] The United States National Library of Medicine (2022). Drug label information of irinotecan hydrochloride injection from Sagent Pharmaceuticals. Available at: https://dailymed.nlm.nih.gov/dailymed/drugInfo.cfm?setid=caab50e6-0dad-4ddd-9a6c-96cc2b87aee7#section-2.1 (Accessed August 22, 2023).

[B34] U. S. Food and Drug Administration (2005). Guidance for industry: estimating the maximum safe starting dose in adult healthy volunteer. Available at: https://www.fda.gov/media/72309/download (Accessed August 22, 2023).

[B35] Wang L. Zhang Q. Xu Y. Zhang R. Zhao Q. Zhang Y. (2023). Ginsenoside Rb1 suppresses AOM/DSS-Induced colon carcinogenesis. Anticancer Agents Med. Chem. 23, 1067–1073. 10.2174/1871520623666230119092735 36655530

[B36] Wu F. Lai S. Feng H. Liu J. Fu D. Wang C. (2022). Protective effects of protopanaxatriol saponins on ulcerative colitis in mouse based on UPLC-Q/TOF-MS serum and colon metabolomics. Molecules 27, 8346. 10.3390/molecules27238346 36500439 PMC9738265

[B37] Yang M. Li W. Xie J. Wang Z. Wen Y. Zhao C. (2021). Astragalin inhibits the proliferation and migration of human colon cancer HCT116 cells by regulating the NF-κB signaling pathway. Front. Pharmacol. 12, 639256. 10.3389/fphar.2021.639256 33953676 PMC8091521

[B38] Yu L. Zhou Y. Yang Y. Lu F. Fan Y. (2017). Efficacy and safety of compound kushen injection on patients with advanced colon cancer: a meta-analysis of randomized controlled trials. Evid.-based Complement. Altern. Med. 2017, 7102514. 10.1155/2017/7102514 PMC5702402 29259647

[B39] Zhang J. Wang X. Li H. Chen C. Liu X. (2022). Immunomodulatory effects of chicken broth and histidine dipeptides on the cyclophosphamide-induced immunosuppression mouse model. Nutrients 14, 4491. 10.3390/nu14214491 36364753 PMC9659005

[B40] Zuo Z. Jia J. Li H. Shi R. Wang D. Zeng K. (2022). Adjuvant effects of Chinese medicinal tonics on gastric, liver, and colorectal cancers-OMICs-based contributions to understanding their mechanism of action. Front. Pharmacol. 13, 986765. 10.3389/fphar.2022.986765 36523499 PMC9746692

