# Supplementary material for: Evaluation of pharmacological and pharmacokinetic herb-drug interaction between irinotecan hydrochloride injection and Kangai injection in colorectal tumor-bearing mice and healthy rats
Source: Front Pharmacol. 2023 Nov 29;14:1282062. doi: 10.3389/fphar.2023.1282062 (PMC10716275; doi:10.3389/fphar.2023.1282062)
Supplement: Supplementary file 1 [file Table1.DOCX]

Supplementary Material

**Table S1.** The calibration curves and LLOQ for the key components of KA injection in rat plasma

| Analytes | Regression equation | Linear range (ng/mL) | R | LLOQ (ng/mL) |
| --- | --- | --- | --- | --- |
| Oxymatrine | y = 7.30 × 10^4^x – 70.8 × 10^4^ | 10-4000^#^ | 0.9962 | 10 |
| Matrine | y = 9.31 × 10^4^x – 22.2 × 10^4^ | 10-4000 | 0.9996 | 10 |
| Ginsenoside Rb1 | y = 13.6 × 10^4^x – 82.6 × 10^4^ | 10-4000 | 0.9951 | 10 |
| Ginsenoside Rg1 | y = 34.5 × 10^4^x – 70.0 × 10^4^ | 10-4000 | 0.9957 | 10 |
| Ginsenoside Re | y = 18.1 × 10^4^ x – 84.4 × 10^4^ | 10-4000 | 0.9940 | 10 |
| Astragaloside IV | y = 8.78 × 10^4^ x – 53.2 × 10^4^ | 10-4000 | 0.9908 | 10 |

^#^ The samples presented in a concentration exceeding the range of quantification were diluted in the blank matrix to bring the concentrations into the range.

**Table S2.** Accuracy and precision for the key components of KA injection in rat plasma

| Analytes | Added concentration (ng/mL) | Intra-batch (n = 6) | | Inter-batch (n = 18) | |
| --- | --- | --- | --- | --- | --- |
|  |  | RSD (%) | RE (%) | RSD (%) | RE (%) |
| Oxymatrine | 20 | 13.2 | -3.54 | 12.4 | -1.81 |
|  | 1000 | 13.9 | 6.61 | 12.4 | 2.40 |
|  | 3000 | 11.6 | -1.99 | 8.34 | -5.59 |
| Matrine | 20 | 12.8 | -7.57 | 12.6 | -4.15 |
|  | 1000 | 10.2 | 0.37 | 11.2 | -2.02 |
|  | 3000 | 10.5 | -10.7 | 10.6 | -10.1 |
| Ginsenoside Rb1 | 20 | 10.7 | 7.45 | 11.2 | 3.04 |
|  | 1000 | 8.85 | -3.53 | 13.0 | -2.95 |
|  | 3200 | 3.33 | -5.32 | 12.6 | 2.32 |
| Ginsenoside Rg1 | 20 | 6.12 | -6.18 | 13.7 | -2.96 |
|  | 1000 | 8.09 | -4.13 | 8.52 | -5.04 |
|  | 3200 | 4.31 | -13.4 | 3.68 | -13.5 |
| Ginsenoside Re | 20 | 10.7 | -0.06 | 13.0 | 1.21 |
|  | 1000 | 13.5 | -9.26 | 10.7 | -4.39 |
|  | 3200 | 3.81 | -4.92 | 5.82 | -10.1 |
| Astragaloside IV | 20 | 13.9 | -8.56 | 14.6 | -6.61 |
|  | 1000 | 12.2 | -5.78 | 12.7 | -3.48 |
|  | 3200 | 3.11 | 6.42 | 8.93 | -4.40 |

**Table S3.** Matrix effect and recovery for the key components of KA injection in rat plasma (n = 6)

| Analytes | Added concentration (ng/mL) | Matrix effect | | Recovery | |
| --- | --- | --- | --- | --- | --- |
|  |  | (Mean ± SD%) | RSD (%) | (Mean ± SD%) | RSD (%) |
| Oxymatrine | 20 | 104 ± 11 | 11.0 | 79.8 ± 5.6 | 7.07 |
|  | 1000 | 104 ± 4 | 3.80 | 73.6 ± 9.4 | 12.8 |
|  | 3000 | 100 ± 3 | 3.17 | 95.8 ± 4.7 | 4.88 |
| Matrine | 20 | 87.3 ± 3.4 | 3.86 | 137 ± 17 | 12.7 |
|  | 1000 | 98.6 ± 5.1 | 5.14 | 106 ± 10 | 9.79 |
|  | 3000 | 97.0 ± 2.9 | 2.99 | 100 ± 2 | 1.85 |
| Ginsenoside Rb1 | 20 | 95.5 ± 9.0 | 9.38 | 75.9 ± 4.1 | 5.40 |
|  | 1000 | 112 ± 6 | 5.46 | 81.1 ± 2.2 | 2.65 |
|  | 3200 | 112 ± 4 | 3.65 | 74.5 ± 5.3 | 7.13 |
| Ginsenoside Rg1 | 20 | 69.6 ± 2.2 | 3.23 | 71.5 ± 8.9 | 12.5 |
|  | 1000 | 67.0 ± 3.9 | 5.79 | 76.1 ± 2 | 2.64 |
|  | 3200 | 60.3 ± 2.1 | 3.49 | 75.6 ± 5.2 | 6.89 |
| Ginsenoside Re | 20 | 91.1 ± 4.7 | 5.15 | 67.0 ± 5.4 | 8.03 |
|  | 1000 | 70.4 ± 4.8 | 6.88 | 75.5 ± 2.0 | 2.58 |
|  | 3200 | 64.0 ± 1.8 | 2.87 | 73.9 ± 5.4 | 7.26 |
| Astragaloside IV | 20 | 93.1 ± 12.8 | 13.7 | 75.9 ± 11.2 | 14.7 |
|  | 1000 | 101 ± 5 | 5.26 | 82.7 ± 3.2 | 3.90 |
|  | 3200 | 100 ± 4 | 4.12 | 75.0 ± 3.3 | 4.50 |
| Donepezil (IS) | 200 | 101 ± 2 | 2.25 | 95.3 ± 1.5 | 1.54 |
| Digoxin (IS) | 20000 | 59.2 ± 1.9 | 3.29 | 78.7 ± 2.4 | 3.10 |

**Table S4.** Stability for the key components of KA injection in rat plasma (n = 3)

| Analytes | Added concentration | Room temperature for 4 h | | Autosampler for 24 h | | Three freeze-thaw cycles | | Frozen for 10 d | |
| --- | --- | --- | --- | --- | --- | --- | --- | --- | --- |
|  | (ng/mL) | RSD (%) | RE (%) | RSD (%) | RE (%) | RSD (%) | RE (%) | RSD (%) | RE (%) |
| Oxymatrine | 20 | 14.5 | -2.77 | 4.35 | -5.08 | 6.92 | -1.57 | 3.82 | -7.31 |
|  | 3000 | 1.67 | -10.4 | 0.74 | -11.5 | 3.37 | -8.53 | 1.42 | -8.33 |
| Matrine | 20 | 5.19 | 4.58 | 2.07 | 2.71 | 3.00 | 5.23 | 0.86 | 0.54 |
|  | 3000 | 1.46 | -5.79 | 1.56 | -7.28 | 2.16 | -2.84 | 2.00 | -2.26 |
| Ginsenoside Rb1 | 20 | 4.73 | 9.85 | -3.18 | 9.19 | -8.70 | 9.77 | -7.29 | 8.20 |
|  | 3200 | -6.54 | 3.14 | -5.07 | 3.72 | -4.74 | 5.64 | -3.53 | 4.43 |
| Ginsenoside Rg1 | 20 | 11.7 | 6.74 | 7.50 | 10.5 | -5.69 | 5.80 | -0.12 | 11.7 |
|  | 3200 | -13.7 | 2.69 | -12.8 | 4.20 | -8.09 | 6.49 | -11.8 | 2.64 |
| Ginsenoside Re | 20 | -15.0 | 2.30 | -4.99 | 6.71 | -4.88 | 5.78 | 0.26 | 8.95 |
|  | 3200 | -14.1 | 3.64 | -12.2 | 5.20 | -7.83 | 7.05 | -10.8 | 1.89 |
| Astragaloside IV | 20 | -2.18 | 5.20 | -7.25 | 6.37 | -11.7 | 12.0 | -13.1 | 1.47 |
|  | 3200 | -10.4 | 3.36 | -9.21 | 3.56 | -5.47 | 5.32 | -6.89 | 1.34 |
